# Community evaluation of the physical and insecticidal durability of DuraNet® Plus, an alpha-cypermethrin and piperonyl butoxide incorporated mosquito net: protocol for a multi-country study in West, Central and East Africa

**DOI:** 10.1186/s13690-023-01217-w

**Published:** 2023-11-20

**Authors:** Abel Agbevo, Idelphonse Ahogni, Benjamin Menze, Patrick Tungu, Elison E. Kemibala, Renaud Govoetchan, Charles Wondji, Germain Gil Padonou, Corine Ngufor

**Affiliations:** 1 Centre de Recherches Entomologiques de Cotonou (CREC), Cotonou, Benin; 2 Panafrican Malaria Vector Research Consortium (PAMVERC-BENIN), Cotonou, Benin; 3 London School of Hygiene and Tropical Medicine (LSHTM), London, UK; 4 Centre for Research in Infectious Diseases (CRID) ), Yaoundé, Cameroon; 5 Vector Control Training Centre (VCTC), Muheza, Tanzania; 6 National Institute for Medical Research (NIMR), Amani Research Centre, Tanga, Tanzania; 7 Liverpool School of Tropical Medicine, Liverpool, UK

**Keywords:** Durability, LLIN, Insecticide treated net, ITN, Piperonyl Butoxide, PBO, Malaria, Insecticide, Bioefficacy, DuraNet® Plus, DuraNet®

## Abstract

**Background:**

Pyrethroid-PBO nets have demonstrated improved impact against clinical malaria transmitted by pyrethroid resistant mosquito vectors and are being scaled up across Africa. However very little is known about their physical and insecticidal durability under operational conditions. This study will investigate the attrition, fabric integrity, insecticide content and bioefficacy of DuraNet® Plus, a new WHO prequalified alphacypermethrin and PBO incorporated net developed by Shobikaa Impex Private Limited over 3 years of field use in communities in Benin, Cameroon and Tanzania.

**Methods:**

The study will be conducted in parallel in selected villages in Zakpota District in Benin, Mbalmayo, District in Cameroon and Muheza District in Tanzania. In each country, ~ 1800 households will be recruited and randomised to receive DuraNet® Plus or DuraNet® (a WHO prequalified alphacypermethrin-only ITN). Follow up surveys will be performed at 1 month post distribution to investigate adverse events and subsequently every 6–12 months to assess ITN attrition and fabric integrity following standard WHO procedures. A second cohort of nets will be withdrawn every 6–12 months and assessed for alpha-cypermethrin and PBO content and for entomological activity in laboratory bioassays (cone bioassays and tunnel tests). Alpha-cypermethrin bioefficacy will be monitored using the susceptible Anopheles gambiae Kisumu strain in cone bioassays while PBO bioefficacy will be monitored using pyrethroid resistant strains with overexpressed P450 enzymes in tunnel tests to determine the proportion of efficacious nets (≥ 95% knockdown, ≥ 80% mortality or ≥ 90% blood feeding inhibition in tunnels) at each time point. Nets withdrawn at 12, 24 and 36 months from each country will also be tested in experimental hut trials against wild free-flying pyrethroid resistant Anopheles gambiae sl in Côvè Benin to investigate the superiority of DuraNet® Plus over DuraNet® at each time point under semi field conditions.

**Conclusion:**

This large-scale multi country trial will provide useful information on the durability of a pyrethroid-PBO net (DuraNet® Plus) in 3 different regions in sub-Saharan Africa. The methods proposed for bioefficacy testing could also contribute towards the development of new standardised guidelines for monitoring the insecticidal efficacy of pyrethroid-PBO nets under operational conditions.

## Background

Insecticide treated nets (ITNs) contributed > 80% to the decline in malaria burden observed in endemic countries between the years 2000 and 2015 [1]. However, progress against malaria has stalled in recent years attributable to several factors including the increasing intensity of resistance to pyrethroids; the insecticide of choice used on ITNs [2, 3].

Pyrethroid-piperonyl butoxide (pyrethroid-PBO) ITNs were developed to help maintain the effectiveness this intervention against malaria vectors that have developed resistance to pyrethroids [4]. The PBO component of the net inhibits the activity of mosquito enzymes that metabolise pyrethroids resulting in higher levels of mosquito mortality with pyrethroid-resistant mosquitoes. Community randomized-controlled trials in East Africa, have demonstrated an improved epidemiological impact against clinical malaria in communities receiving pyrethroid-PBO nets compared to standard pyrethroid-only nets [5, 6]. Based on these findings, and several experimental hut trials across Africa, the World Health Organisation (WHO) issued a conditional recommendation for their deployment in areas where vectors have developed resistance to pyrethroids [7]. This has been followed by an increased uptake of pyrethroid-PBO nets across Africa in recent years [8].

To be considered long-lasting, new ITNs are expected to demonstrate physical and insecticidal durability under operational conditions lasting at least 3 years [9–11]. Recent modelling studies have however estimated a much shorter median ITN retention time of 1.64 years across Africa [12]. ITN durability is also reported to vary from one community to another influenced by several location specific factors including, usage by householders, perceived efficacy, fabric integrity, environmental conditions etc. [11, 13]. While the WHO has recommended standardised procedures for assessing the attrition, fabric integrity, insecticidal content and bioefficacy of ITNs over 3 years of community use [9, 11], there is very limited information on the durability of pyrethroid-PBO ITNs. This is partly attributable to the fact that existing WHO ITN durability guidelines were largely tailored towards pyrethroid-only nets. Effective assessment of the durability of pyrethroid-PBO ITNs will require the development of suitable standardised methods and procedures for monitoring the bioefficacy of both active ingredients on these nets over years of field use.

DuraNet® Plus is an alphacypermethrin and PBO incorporated polyethylene net developed by Shobikaa Impex Private Limited. It was recently added to the WHO list of prequalified ITNs [14] based on its improved performance compared to pyrethroid-only nets in experimental hut trials conducted across sub-Saharan Africa (unpublished data). To facilitate programmatic and procurement decisions around the uptake of pyrethroid-PBO nets for malaria control, studies comparing the durability of DuraNet® Plus and other PBO nets to standard pyrethroid-only nets are necessary. This protocol describes a two-arm trial to evaluate the durability of DuraNet® Plus in terms of survivorship, fabric integrity, chemical content, bioefficacy, and user acceptance under field conditions in communities in Benin, West Africa, Cameroon, Central Africa, and Tanzania, East Africa, compared to DuraNet®, a WHO prequalified alpha-cypermethrin ITN. The study will be conducted in accordance with existing WHO guidelines for the evaluation of the durability of ITNs under community use [9] with modifications to enable assessment of the insecticidal durability of the PBO component of DuraNet® Plus.

## Study aims

### Overall aim

To assess the physical and insecticidal durability of an alpha-cypermerthrin and PBO ITN (DuraNet® Plus®) compared to a standard alphacypermethrin only net (DuraNet®) over 3 years of operational use in communities in Benin, Cameroon and Tanzania.

### Secondary aim

- ▪ To determine the survivorship and fabric integrity of DuraNet® Plus net compared to a standard alpha-cypermethrin-only net (DuraNet®) over 3 years of household use in Benin, Cameroon and Tanzania.
- ▪ To investigate insecticidal activity and chemical content of DuraNet® Plus net compared to DuraNet® over 3 years of household use in Benin, Cameroon and Tanzania.
- ▪ To document adverse events, community perception and user-acceptability of DuraNet® Plus and DuraNet® over 3 years of household use in Benin, Cameroon and Tanzania.
- ▪ To investigate ITN washing habits and washing frequency over 3 years of household use in Benin, Cameroon and Tanzania.

## Materials and methods

### Study arms

This study consists of two arms evaluating the durability of DuraNet® Plus compared to DuraNet® A description of the two ITN types is provided below:

1. DuraNet® Plus is WHO prequalified pyrethroid-PBO mixture 150 dernier net made of monofilament high density polyethylene (HDPE). It is incorporated with a mixture of alpha-cypermethrin at 6.0 g of active ingredient/kg and Piperonyl Butoxide (PBO) at 2.2 g of active ingredient/kg. It has a mesh size of 20 holes/cm^2^ and a fabric weight of 45 g/m^2^.
2. DuraNet® is a WHO prequalified standard pyrethroid-only 150 dernier net also made of mono-filament high density polyethylene (HDPE). It is incorporated with alpha-cypermethrin at 5.8 g of active ingredient/kg. It has a mesh size of 20 holes/cm2 and a fabric weight of 45 g/m^2^. DuraNet® will serve as the reference net against which DuraNet® Plus will be compared.

DuraNet® Plus and DuraNet® are manufactured by Shobikaa Impex pvt Ltd (Karur, Tamil Nadu 639,006, and India).

### Study setting

The study will be conducted in selected malaria endemic villages in 3 different countries across sub-Saharan Africa: Benin, Cameroon and Tanzania. The countries have been selected to provide a suitable representation in the different regions in the area, West (Benin), Central (Cameroon) and East (Tanzania) Africa. Villages have been selected based on road accessibility, willingness to participate, availability of enough households, history of high ITN usage and proximity to the laboratory and insectary facilities of the participating institutional partners. A description of study area chosen for each country is provided in Table 1 below:

**Table 1:** Description of study sites in Benin, Cameroon and Tanzania

Characteristics	Benin	Cameroon	Tanzania
Study villages	Agonkanme, Zantata Centre, Doutin and Agondokpoe	Ekoumeyeck and Agonfeme	Kibaoni, Magila, Misongeni and Ubembe
Estimated population size	~ 11,000	~ 6000	~ 7000
District	Zakpota District in Zou Department	Mbalmayo Districts in Central Region	Muheza district in Tanga Region
Geographical Coordinates	Latitudes 7° 13′ 49″ North Longitudes 2° 12′ 55″ East	Latitude: 3° 31′ 12″ North, Longitude: 11° 30′ 44″ East	Latitude: 5° 10′ 0.012" South. Longitude: 38° 46′ 59.988" East
Climate	Tropical humid climate Annual rainfall 1003.4mm and temperature of 27.1°C	Tropical savannah climate with wet and dry season. Annual rainfall 2402.8mm and temperature 26.5°C	Tropical savannah climate with wet and dry season Annual rainfall 293.6mm and temperature 23.7°C
Main vector population	*Anopheles gambiae sl.* resistant to pyrethroids	*Anopheles gambiae sl.* and *Anopheles funestus* resistant to pyrethroids	*Anopheles gambiae ss.* and *Anopheles funestus* resistant to pyrethroids
Typical economical activities	Agriculture and livestock	Agriculture, fishing and livestock	Agriculture, and livestock

### Study design outline

**Fig. 1:**
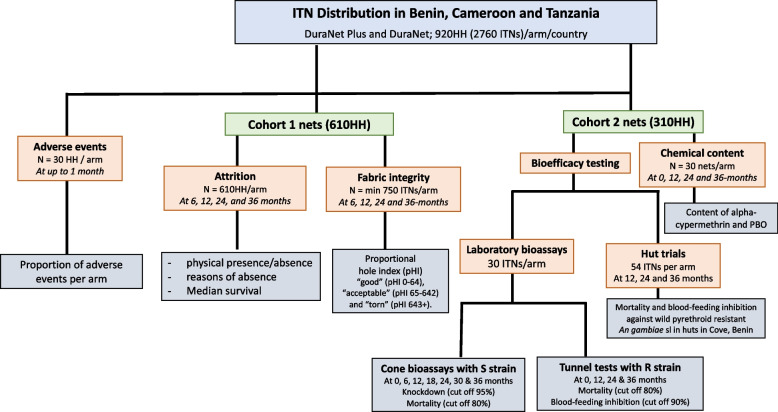
Flow chart of trial activities and outputs for monitoring the physical and insecticidal durability of DuraNet Plus in Benin, Cameroon and Tanzania. *Two cohorts of nets will be followed per study arm; cohort 1 nets will be assessed for ITN attrition and fabric integrity while cohort 2 nets will be withdrawn (and replaced) and assessed for bioefficacy and chemical content*

The study will be conducted in a minimum of 1800 households in each country to be randomly assigned to the two study arms. Nets will be distributed to each household and followed prospectively. Before net distribution, a baseline census will be carried out in the selected study villages to collect demographic information and socio-economic characteristics of the participating households. This will be followed by a net distribution campaign after which a series of follow-up surveys will be performed to assess adverse events, ITN survivorship, fabric integrity, insecticidal efficacy and chemical content over a period of 36 months. Figure 1 provides a summary of the surveys and the different studies to be performed at each timepoint.

### Randomisation, blinding and sample size calculations

Households will be the unit of randomisation of this study. Participating households will be blinded to the type of ITN they received while data collectors will be blinded to the allocation of households to the study arms. Study nets (DuraNet® Plus and DuraNet®) will be identical in appearance. Two cohorts of nets of each ITN type will be followed; cohort 1 to assess ITN survivorship, attrition and Fabric integrity and cohort 2 for nets to be withdrawn for assessment of bioefficacy and insecticide content. A sample size of 510 households per study arm was found sufficient to detect a 7 points difference in ITN functional survival rate over 3 years between households receiving DuraNet® Plus and those receiving DuraNet® with 80% power, assuming a baseline 3-year survival rate of 25%. This sample size was increased by 100 households to account for loss to follow up over time. To account for nets to be withdrawn for bioefficacy studies, hut trials and insecticidal content, an additional 310 households per arm will be included in the study, hence a total of 920 households will be recruited per study arm at each site. It is estimated that about 3 nets will be required per household therefore the study will require 2,760 nets of each ITN type at each study site.

### Community sensitisation and baseline census

A series of community sensitisation meetings will be held in selected villages in each study site prior to the baseline surveys to ensure community participation. The study procedure and its objectives will be explained to leaders of all participating villages to ensure their cooperation with the study team. The meetings will be conducted in the local language of each community to ensure full participation. After the community sensitisation meetings, a baseline census will be conducted in all consenting households in the selected villages for each study site using a structured questionnaire. The census will provide a framework for allocation of study nets and for sampling at follow-up. The following information will be recorded: name of the village, name of the head of the household, number of household members with details of adults and children living in the house, number of nets already in the household, number of sleeping places and the global positioning system (GPS) coordinates will be recorded to assist in identifying houses during follow-up. Each house will be assigned a unique identifier which will be marked on the door of the house during the survey using indelible ink.

### ITN distribution and net master lists

The baseline census data will be used to randomly allocate households to each study arm using a random number generation scheme. The study team will then visit all participating households to provide vouchers for collection of study ITNs at a central point in each village. Where possible, households will be visited door-to-door to ensure that they receive their allocated net. Each ITN will be marked with a unique identifier before being provided to the household. The unique identifier will contain the household number, a unique number for the ITN, the ITN type and the cohort (cohort 1 and 2) to which the net has been assigned. Using these unique identifiers, net master lists will be generated, and these will contain a unique identification for each study net available in each household and separated by cohort to avoid any risk of destructive sampling of nets from households in cohort 1. As described previously, nets in the cohort 1 master list will be followed for attrition and fabric integrity while nets in the cohort 2 master lists will be withdrawn (and replaced with new nets of same type) for bioefficacy and insecticidal content studies.

### Follow up surveys

#### Follow-up for ITN hang-up and adverse events

Between 1 week and up to 1 month post-distribution, sleeping places in houses in the net sampling lists (cohort 1 and cohort 2) will be observed to ensure that study nets have been set up. Where necessary, study teams will assist participants in hanging up their nets. The nets available in the house will also be checked to ensure that they match with what is available in the net sampling list which was generated after distribution and corrections will be made as necessary. During these visits, householders will be encouraged to use the study nets over any other nets available with them. Using a questionnaire, the adverse effects related to net usage will be investigated in 30 randomly selected households in each study arm at each study site. Heads of households will be questioned on any perceived adverse events including sneezing, itching, headache, numbness, eye discharge, any unpleasant smells and nausea in line with WHO guidelines. Participants will be advised to seek medical care from the nearest health facility should the symptoms persist.

#### Follow-up for ITN attrition

Net attrition rate in households will be measured by recording the physical presence of the net at 6-, 12-, 24- and 36-months post ITN distribution. All households in cohort 1 will be visited and nets assessed for attrition at each timepoint by recording the physical presence/absence of each net attributed to the household. Where a net is not found, the householder will be questioned about the reason for the loss of the net e.g. given away, sold, stolen, worn out and disposed of etc. Any study nets which have never been used, will be recorded and excluded from the analysis. ITNs lost due to wear and tear will be considered as lost due to attrition, and those lost because they were stolen, sold or given away to others, will be considered as lost to follow-up. During these surveys, a questionnaire adapted from WHO guidelines will be used to collect data on the number of washes and washing practices for the nets found in each household. The household surveys will be conducted by an experienced socio-anthropologists.

#### Follow-up for ITN fabric integrity

Fabric integrity (hole index) and condition will be observed on all nets of cohort 1 at 6-, 12-, 24- and 36-months post distribution. Where the minimum number (250) of nets for fabric integrity is not attainable from the longitudinal cohort (cohort 1) due to loss, ITNs sampled destructively from the same clusters for bio-efficacy would first be examined for fabric integrity prior to the bio-efficacy studies. The survey team will inspect the nets outside in broad day light to determine the hole index using a portable frame over which the net can be draped during inspection. The nets will be returned to the family right after the inspection. Hole number and size will be assessed on each ITN panel using hole assessment sheet and classified into 4 sizes (size 1: 0.5-2 cm, Size 2: 2–10 cm, Size 3: 10-25 cm, and size 4: > 25 cm). Physical integrity will be measured as the proportionate hole index (pHI), categorized based on recommended cut-off points into “good” condition (pHI 0–64), “acceptable” condition (pHI 65–642) and “torn” (pHI 643 +).

### Bioefficacy studies and chemical analysis

A separate subsample of nets belonging to cohort 2 will be randomly selected per arm for bioefficacy studies and chemical analysis. Nets of each type (DuraNet® Plus and DuraNet®) will be destructively sampled from households at 6, 12, 18-, 24-, 30- and 36-months post-distribution and replaced with new nets of the same type (brand) at each sampling point. They will be subjected to chemical analysis to monitor alphacypermethrin and PBO content and to laboratory bioassays (WHO cone bioassays and tunnel tests where necessary) and experimental hut trials to monitor entomological efficacy against mosquito vectors over time. For laboratory bioassays, net pieces measuring 30cmx30cm will be cut from each net following WHO guidelines [9]. The number of whole ITNs of each type to be subjected to each type of study at each timepoint is summarised in Table 2 below.

**Table 2:** Number of DuraNet® Plus and DuraNet® ITNs to be sampled for laboratory assays, chemical analysis and hut trials at each study site

Months post ITN distribution	No of ITNs for Laboratory assays	No. of pieces per ITN	Total number of pieces for bioassays	No. of pieces for chemical analysis^a^	ITNs for hut trials^b^
0	30	5	150	150	-
6	30	4	120	-	-
12	30	4	120	120	54
18	30	4	120	-	-
24	30	4	120	120	54
30	30	4	120	-	-
36	50	4	200	200	54
**Total**	**230**		**950**	**590**	**162**

^a^ Net piece preserved for chemical analysis will be obtained from adjacent positions on the same ITNs cut for bioassays. These net pieces would measure 30cmX30cm

^b^The hut trials will be performed only in Benin but will include nets from all study sites

#### Chemical analysis

The net pieces preserved for chemical analysis at 0, 12, 24 and 36 months (590 pieces per net type) will be wrapped in Aluminum foil and stored at 4^0^c (± 2°c) and afterwards shipped for chemical content analysis to International Institute of Biotechnology and Toxicology (IIBAT), a GLP laboratory in India for detection of alphacypermethrin and PBO content using high performance liquid chromatography. Net pieces will be shipped within 3 months after collection.

#### WHO cone bioassays and tunnel tests

Cone bioassays and tunnel tests will be performed at research partner institutions in each study country (Centre de Recherche Entomologique de Cotonou in Benin, Centre for Research in Infectious Diseases, Cameroon, and Vector Control Training Centre in Tanzania). To monitor the bioefficacy of alphacypermethrin in DuraNet® Plus and DuraNet®, ITN pieces obtained from each field collected net will be tested in standard WHO cone bioassays. One hundred (100) unfed 3–5 days old mosquitoes of the susceptible *Anopheles gambiae* Kisumu strain will be exposed for 3 min to each ITN in replicates of 5 mosquitoes per cone and 2 cones per net piece. Knockdown will be recorded after 60 min and mortality rates after 24 h. Comparison will be made to a new unused net of each ITN type. ITNs which fail to achieve WHO efficacy criteria in cone bioassays (pooled knock-down is ≥ 95% and/or pooled 24 h mortality is ≥ 80%) will be subjected to tunnel tests. For tunnel tests, 100 unfed 5–8 days old susceptible Kisumu mosquitoes will be exposed overnight to one ITN piece from each failed net and mortality (after 24 h) and blood-feeding inhibition rates measured following standard WHO protocols. The proportion of ITNs of each type passing criteria for alpha-cypermethrin bioefficacy in cone bioassays (≥ 95% knockdown or ≥ 80% mortality) and/or tunnel tests (≥ 80% mortality or ≥ 90% blood-feeding inhibition) will be determined for each study site. All cone bioassays will be performed at 27 ± 2 °C and 75% ± 10%

To monitor the bioefficacy of PBO in DuraNet® Plus, tunnel tests will be performed using 1 net piece from ITNs withdrawn for bioassays at 12, 24 and 36 months using the pyrethroid resistant *An. coluzzi* Akron strain (BEI resources) for Benin and Cameroon and the pyrethroid resistant *An gambiae ss* Zenet strain in Tanzania. Both strains exhibit intense resistance to pyrethroids mediated by high kdr frequencies and overexpressed P450 enzymes evidenced by increased mortality with alpha-cypermethrin following PBO pre-exposure in susceptibility bioassays. They will be fully characterised prior to each round of laboratory bioassays. The proportion of DuraNet® Plus ITNs inducing ≥ 80% mortality of the pyrethroid resistant strain in tunnel tests will also be determined for each study site. Comparison will be made with a new unused DuraNet® Plus at each timepoint. All tunnel tests will run overnight between 18:00 and 09:00 at 27 ± 2 °C and 75% ± 10% relative humidity.

#### Experimental hut trials

To further assess the entomological superiority of DuraNet® Plus over DuraNet®, experimental hut trials will be performed with field collected ITNs at 12-, 24- and 36-months post ITN distribution from each country at the Cove experimental hut station in Benin against wild free-flying pyrethroid-resistant *Anopheles gambiae* sl. Fifty-four (54) DuraNet® Plus and 54 DuraNet® nets from all three countries will be tested together in experimental huts at each annual timepoint. Comparison will be made with new unused nets of each type. The hut trials will consist of the following 9 treatments:

1. Untreated net – 6 replicates
2. DuraNet® (new unused) – 6 replicates
3. DuraNet® Plus (new unused) – 6 replicates
4. DuraNet® Benin (12-, 24- or 36-months field net) – 54 replicates
5. DuraNet® Cameroon (12-, 24- or 36-months field net) – 54 replicates
6. DuraNet® Tanzania (12-, 24- or 36-months field net) – 54 replicates
7. DuraNet® Plus Benin (12-, 24- or 36-months field net) – 54 replicates
8. DuraNet® Plus Cameroon (12-, 24- or 36-months field net) – 54 replicates
9. DuraNet® Plus Tanzania (12-, 24- or 36-months field net) – 54 replicates

Each trial will last 9 weeks and will involve 9 consenting human volunteers sleeping in huts from dusk to dawn 6 days of each week of the trial. To allow assessment of blood-feeding inhibition, all unused nets will be given 6 holes each measuring 4cmx4cm in accordance with WHO guidelines [9]. Treatments will be rotated each week using a randomised Latin Square Design while sleepers will be rotated daily to control for hut position effect and sleeper attractiveness to mosquitoes respectively. On the 7^th^ day of each week, huts will be cleaned and aired in preparation for the next rotation cycle. The trials will run for a total of 54 nights and replicate nets collected from the field will be rotated between huts on successive nights (one net per night). The efficacy of each ITN hut treatment at each timepoint will be measured in terms of the proportion of mosquitoes dead after 24 h, proportion exiting into the veranda trap and the reduction in blood-feeding relative to the control untreated nets. The intensity of resistance to pyrethroids in the vector population at the hut site and the involvement of mixed function oxidases in pyrethroid resistance will be assessed during each experimental hut trial following standard WHO susceptibility bioassay guidelines [15].

### Data management and analysis

Household data collected during the census and ITN follow up surveys will be captured on electronic forms using smartphones installed with OpenDataKit (ODK) Collect in Benin and by paper forms in Cameroon and Tanzania. Data from the surveys at 12, 24, and 36 months will be used to calculate attrition, functional survival and median survival time using Kaplan–Meier estimators. For functional survival, nets reported as given away, sold, or stolen will be excluded from the analysis. Negative binomial regression will be used to compare hole surface area between DuraNet® Plus and DuraNet®. Data on WHO bioassays and tunnel tests will be obtained from 30 nets of each product type sampled at each time point. A chi-squared test will be used to assess the proportion of nets of each ITN type passing the WHO criteria for alphacypermethrin and PBO bioefficacy based on combined cone and tunnel tests. Proportional outcomes (mortality, blood-feeding, exophily) from the experimental hut trials will be compared at each time point using mixed effects logistic regression, with huts, sleepers, net replicate and nightly observational error included as random-effects while numerical outcomes (entry) will be compared with negative binomial regression. A separate model will be fitted for each outcome to determine whether there is a significant change in improved mosquito mortality achieved DuraNet® Plus compared to DuraNet® over time and between the three countries. All analysis will be performed on STATA version 17.

### Ethical considerations

Ethical approval for this study will be sought from the national ethics review committee of the Ministry of Health in Benin, Cameroon and Tanzania. Prior to any project activities, village and hamlet leaders will be invited to sensitization sessions conducted by district health officers and written informed consent will be sought from the local leader before starting data collection. Heads of households involved in the study will give informed consent prior to their participation in the study. The consent form will be written in French for Benin and Cameroon abd Swahili for Tanzania and will indicate the purpose of the study, the procedures, risks and benefits, that participation is completely voluntary, and that they may withdraw at any time. Where necessary, the consent form will be explained to them in their local language by a trained interpreter. Participants will be asked to sign the consent form in duplicate, one will be kept by the project and they will keep the other. Where the individual is unable to read or write, their fingerprint will be taken, and a signature obtained from a witness to the informed consent procedure. All personal data will be anonymised prior to data processing. Written informed consent will also be obtained from all human volunteer sleepers for experimental hut trials prior to participation. Sleepers will be offered a free course of chemoprophylaxis spanning the duration of the study and 4 weeks following its completion to mitigate malaria infection risk. Approval for use of guinea pigs for tunnel tests in Benin has been obtained from the LSHTM Animal Welfare Ethics Review Board (Ref: 2020–01). In all study countries, guinea pig colonies will be maintained according to institutional standard operating procedures (SOPs) developed in line with relevant national and international regulations governing use of animals for scientific research purposes.

## Discussion

This protocol describes a large-scale study to evaluate the durability of DuraNet® Plus, a new WHO prequalified alphacypermethrin-PBO net compared to an alphacypermethrin-only net (DuraNet®) in communities in 3 different countries across sub-Saharan Africa. While all WHO prequalified pyrethroid-PBO ITNs have at least demonstrated improved entomological efficacy compared to pyrethroid-only nets, their impact under operational conditions will largely depend on their durability and quality. ITN durability studies are useful for planning the replacement of worn-out nets and making programmatic and procurement decisions [10]. Durability can however be influenced by several factors including location specific effects such washing practices, net care, housing structure, climate etc. which can result in significant variations in ITN retention and impact from one community to another [13]. The multi-country approach of our study takes this into account and will provide useful information about how these factors would affect the physical durability of a pyrethroid-PBO net across communities in countries in the three major malaria endemic regions of sub-Saharan Africa.

In addition to their physical durability, novel ITNs with new active ingredients other than pyrethroids, need to be closely monitored for the durability of their bioefficacy under field use. Earlier studies investigating the durability of Olyset® Plus nets (a permethrin-PBO net) in line with existing WHO guidelines, used only pyrethroid-susceptible mosquitoes for monitoring their bioefficacy [16]. However, this approach would likely lead to unreliable results as the impact of PBO will be confounded by the pyrethroid insecticide in the net to which the mosquitoes are susceptible. Effective monitoring of the biological activity of the PBO component in these nets requires a pyrethroid-resistant strain with evidence of metabolic resistance mechanisms as proposed in our study protocol and in recently developed consensus standard operating procedures [17]. This allows for measurement of the additional biological effect of PBO in the net over the pyrethroid. Recognising that mosquito strains used in laboratory bioassays are prone to variability in their insecticide resistance profiles over time, to improve comparability of bioefficacy data across our study sites, we will characterise the strains used in our study prior to each round of bioassays following consensus protocols [18].

A more recent study in Uganda investigating the durability of the bioefficacy of pyrethroid-PBO nets with a pyrethroid-resistant strain with overexpressed P450 enzymes in cone bioassays and wire ball assays demonstrated a substantial decline in PBO bioefficacy over 25 months of field use [19]. However, in contrast to WHO protocols requiring that ITNs which fail to achieve efficacy criteria in cone bioassays are tested in tunnel tests [9], the study nets from the Ugandan trial were not further assessed in tunnel tests which means the durability of the PBO component may have been underestimated. Preliminary studies in Benin have also demonstrated very low ITN pass rates with pyrethroid-PBO nets against pyrethroid-resistant mosquitoes in WHO cone bioassays compared to tunnel tests (unpublished data). Tunnel tests can be more demanding, but they mimic mosquito host seeking behaviour in the presence of the net and an animal bait and thus provide more realistic bioefficacy results compared to cone bioassays. To mitigate this problem and improve the efficiency of the bioassays, in each country, we will monitor the bioefficacy of the PBO component in DuraNet® Plus directly in tunnel tests with a pyrethroid-resistant strain.

ITN physical durability studies and laboratory bioassays may not be sufficiently informative about the levels of personal and community protection against malaria that pyrethroid-PBO nets can provide to users as they age. Experimental hut trials are a suitable and reliable method for investigating the performance of indoor vector control interventions in human occupied household settings against wild free-flying mosquitoes. Recent modelling studies have demonstrated that the performance of ITNs against clinical malaria can be inferred from experimental hut trials [20]. Hence the experimental hut trials with field collected nets against wild free-flying pyrethroid-resistant malaria vectors in Benin in this protocol, will provide more insights into the potential performance of DuraNet® Plus against clinical malaria under operational use in each country. The design will also allow for a direct comparison of the potential impact of these nets at different time points post distribution from the study countries.

One limitation of the study is that the chemical analysis will focus on assessing the total content of each active ingredient in the study nets. While this aligns with current WHO guidelines we note that evaluation of the surface concentration of the active ingredients on ITNs would be more informative as this would provide an indication of their bioavailability for mosquito control as the nets age. We would however explore additional opportunities to assess the surface concentration of the study nets following suitable methods to complement the study.

## Conclusion

This large-scale multi country trial will provide useful information on the durability of a pyrethroid-PBO net in 3 different regions in sub-Saharan Africa. The methods proposed for bioefficacy testing could also contribute towards the development of new standardised guidelines for monitoring the insecticidal efficacy of pyrethroid-PBO nets under operational conditions.

## Status of study

A timeline of the study is provided in Table 3 below. Ethical approval has been obtained in all three study countries. The baseline census has been completed and the study nets distributed. ITN follow up surveys and bioassays with withdrawn nets at 6 months post distribution have been completed. Further follow up surveys are ongoing. The final ITN follow-up survey is expected to be completed in June 2025 and the complete data sets from the multi country should be available by end of 2025.

**Table 3:**
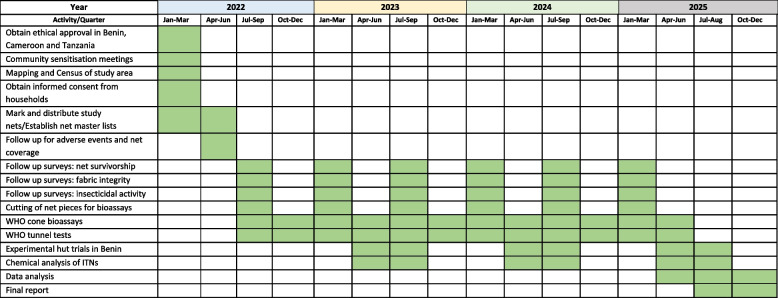
Study timeline for evaluating the durability of DuraNet Plus in Benin, Cameroon and Tanzania.

## Data Availability

Not applicable.
